# C-954-02. Ambient Temperatures and Contact Burns in the Summer Months: A Regional Burn Center’s Experience

**DOI:** 10.1093/jbcr/irag033.134

**Published:** 2026-04-06

**Authors:** Arianna P Lee, Kadee-Kalia Tamashiro, Katrina Waters, Yeng Vue, Nicole M Kopari

**Affiliations:** University of California San Francisco, Fresno, CA; University of California San Francisco, Fresno, CA; University of California San Francisco, Fresno, CA; University of California Merced, Fresno, CA; Leon S. Peters Burn Center, Fresno, CA

## Abstract

**Introduction:**

In many regions, summer ambient temperatures frequently exceed 100°F, heating outdoor surfaces to levels capable of causing partial- and full-thickness contact burns. Prior studies in arid climates have demonstrated seasonal increases in pavement burns. This study characterizes the relationship between ambient temperature and contact burns at a regional burn center.

**Methods:**

We conducted a retrospective review of patients presenting with contact burns from outdoor heated surfaces between June and August of 2022–2024. Burn registry data included demographics, burn characteristics, comorbidities, and outcomes. Daily noon high temperatures were recorded. Descriptive statistics were used to describe demographic and rates of outcomes. For numerical variables, multinomial logistic regression, linear regression, and binary logistic regression were used when comparing temperature to outcomes. Welch's t-test was also used for any binary variable with continuous outcomes. Chi-square test and Fisher's Exact Test were used to analyze categorical variables.

**Results:**

Of 365 total burn encounters, 56 (15.3%) were identified as contact burns from outdoor surfaces. Mean noon high temperatures were 98.5 ± 6.4°F (2022), 94.8 ± 8.5°F (2023), and 99.6 ± 7.4°F (2024). The number of days exceeding 100°F was 48 in 2022, 28 in 2023, and 49 in 2024. Encounters for contact burns increased significantly with rising temperatures (*p*<.001). Temperature did not predict consult acuity, %TBSA, fall-related injury, burn location, or presence of comorbidities. There was no significant association between diabetes or CKD and burn severity or location. However, psychiatric illness was associated with upper extremity burns (*p*=.040). Data collection from 2025 is ongoing, and results are pending.

**Conclusions:**

Extreme summer temperatures significantly contribute to the incidence of outdoor contact burns. Recognition of comorbidities and environmental risk factors may inform prevention strategies, guide public health interventions, and optimize burn center resource allocation as regional temperatures continue to rise.

**Applicability of Research to Practice:**

Understanding seasonal and demographic patterns of contact burns can strengthen patient counseling, shape public health messaging, and prepare burn centers for predictable seasonal surges.

**Funding for the study:**

N/A.

